# Multimorbidität als Prädiktor für eine stationäre Aufnahme in der klinischen Notfall- und Akutmedizin: Monozentrische Clusteranalyse

**DOI:** 10.1007/s00063-024-01180-6

**Published:** 2024-09-11

**Authors:** E. Grüneberg, R. Fliedner, T. Beißbarth, C. A. F. von Arnim, S. Blaschke

**Affiliations:** 1 Zentrale Notaufnahme, Universitätsmedizin Göttingen, Robert-Koch-Str. 40, 37075 Göttingen, Deutschland; 2 Klinik für Geriatrie, Universitätsmedizin Göttingen, Göttingen, Deutschland; 3 Institut für Bioinformatik, Universitätsmedizin Göttingen, Göttingen, Deutschland

**Keywords:** Notaufnahme, Multimorbidität, Disposition, Clusteranalyse, ICD-10-GM-Diagnosen, Emergency department, Multimorbidity, Disposition, Cluster analysis, ICD-10-GM diagnose

## Abstract

**Hintergrund:**

Infolge der demografischen Entwicklung ist ein deutlicher Anstieg von multimorbiden Notfallpatient*innen in der klinischen Notfall- und Akutmedizin in Deutschland zu verzeichnen. Zur Definition operationalisierbarer Kriterien für die Notwendigkeit der stationären Aufnahmeindikation in diesem Patientenkollektiv wurde eine hierarchische Clusteranalyse durchgeführt.

**Methodik:**

In einer retrospektiven, monozentrischen Studie wurden Daten von *n* = 35.249 Notfällen (01/2016–05/2018) analysiert. Multimorbidität (MM) wurde bei Vorliegen von mehr als 5 im Behandlungsverlauf resultierenden ICD-10-GM-Diagnosen definiert. Es erfolgte eine hierarchische Clusteranalyse der zuvor in 112 Subcluster zusammengefassten Diagnosen zur Ermittlung spezifischer Cluster stationärer und ambulanter Fälle.

**Ergebnisse:**

Stationäre Aufnahmen erfolgten bei 81,2 % aller Notfälle (*n* = 28.633). Die Kriterien der MM wurden bei 54,7 % der stationären (*n* = 15.652) und 0,97 % der ambulanten Fälle (*n* = 64) erfüllt. Der Altersunterschied zwischen letzteren war hochsignifikant (68,7/60,8 Jahre; *p* < 0,001).

Durch hierarchische Clusteranalyse wurden für stationär aufgenommene, multimorbide Patient*innen (MP) 13 Cluster mit unterschiedlichen Diagnosen und für ambulante MP 7 Cluster mit vorrangig hämatologischen Malignomen identifiziert. Die Notaufnahmeverweildauer (VWD) stationärer MP war mehr als doppelt so lang (max. 8,3 h) wie die ambulanter MP (max. 3,2 h).

**Schlussfolgerungen:**

Es wurden für MM typische Diagnosekombinationen in Form von *Clustern* identifiziert. Im Vergleich zu monodimensionalen oder kombinierten Diagnosen resultiert durch die statistisch erhobene Clusterbildung eine wesentlich genauere Prognose für die Disposition in der klinischen Notfallversorgung als auch für die leistungsrechtliche Prozesszuordnung.

## Abstract (en)

**Background:**

Parallel to demographic trends, an increase of multimorbid patients in emergency and acute medicine is prominent. To define easily applicable criteria for the necessity of inpatient admission, a hierarchical cluster analysis was performed.

**Methods:**

In a retrospective, single-center study data of *n* = 35,249 emergency cases (01/2016–05/2018) were statistically analyzed. Multimorbidity (MM) was defined by at least five ICD-10-GM diagnoses resulting from treatment. A hierarchical cluster analysis was performed for those diagnoses initially summarized into 112 diagnosis subclusters to determine specific clusters of in- and outpatient cases.

**Results:**

Hospital admission was determined in 81.2% of all ED patients (*n* = 28,633); 54.7% of inpatients (*n* = 15,652) and 0.97% of outpatient cases (*n* = 64) met the criteria for multimorbidity and the age difference between them was highly significant (68.7/60.8 years; *p* < 0.001). Using a hierarchical cluster analysis, 13 clusters with different diagnoses were identified for inpatient multimorbid patients (MP) and 7 clusters with primarily hematological malignancies for outpatient MP. The length of stay in the ED of inpatient MP was more than twice as long (max. 8.3 h) as for outpatient MP (max. 3.2 h.).

**Conclusions:**

The combination of diagnoses typical for MM were characterized as clusters in this study. In contrast to single or combined single diagnoses, the statistically determined characterization of clusters allows for a significantly more accurate prediction of ED patients’ disposition as well as for economic process allocation.

In der klinischen Notfall- und Akutmedizin wurden in der letzten Dekade überwiegend theoretische Modelle mit monodimensionalen Kriterien für die Indikation zur stationären Aufnahme von Notfallpatient*innen entwickelt. Vor dem Hintergrund steigender MM könnten gut operationalisierbare Kriterien für eine stationäre Behandlungsnotwendigkeit dazu beitragen, die VWD in einer Notaufnahme zu minimieren. In diesem Artikel sollen die für MM typischen Cluster für ambulante und stationäre Notfälle präsentiert werden.

## Einleitung

In den zentralen Notaufnahmen (ZNA) in Deutschland zeigte sich in der letzten Dekade ein Fallzuwachs von bis zu 9 % pro Jahr [18]. Angesichts des demografischen Wandels und des medizinischen Fortschritts mit verbesserter Diagnostik und Therapie steigt damit die Anzahl der chronischen Erkrankungen einer einzelnen Person [1]. Diese sog. MM wird von der Leitlinienkommission der Deutschen Gesellschaft für Allgemeinmedizin durch 3 oder mehr chronische Erkrankungen definiert.

Zur Einschätzung der Behandlungsdringlichkeit erfolgt in den ZNA eine strukturierte Ersteinschätzung (sog. Triage) aller Patient*innen mittels international validierter, 5‑stufiger Triagesysteme, wie u. a. dem *Emergency Severity Index *(*ESI*) oder dem *Manchester Triage System *(*MTS;* [5, 16]). Die Leitsymptomatik dieser Patient*innen wird zudem im Rahmen der Triage erhoben und z. B. in Anlehnung an das *Canadian Emergency Department Information System *(*CEDIS*) beschrieben: In diesem CEDIS-Katalog sind 171 Vorstellungsgründe als codierte Diagnosen nach dem Katalog der *International Statistical Classification of Diseases and Related Health Problems *der* World Health Organization* (ICD-10-WHO) hinterlegt [6].

Um eine stationäre Aufnahmeindikation in der ZNA zu begründen, dienten bisher die sog. Kriterien gemäß *German Appropriateness Evaluation Protocol* (G-AEP). Sie beinhalten vor allem bestehende Erkrankungen und deren Schweregrad sowie graduierte Behandlungsformen und soziale Umstände der Notfallpatient*innen, die in Zusammenschau eine stationäre Therapie bedingen. Dabei wird jedoch kaum deren kombiniertes Auftreten berücksichtigt.

In Zeiten überfüllter ZNA mit langer Verweildauer (VWD) war es Ziel dieser Studie, im Rahmen einer monozentrischen, retrospektiven Studie operationalisierbare Kriterien für die stationäre Aufnahmenotwendigkeit multimorbider Notfallpatient*innen zu definieren, um eine raschere Zuordnung der Disposition durchführen zu können.

## Patient*innen und Methodik

Grundlage dieser retrospektiven, monozentrischen Studie waren die Behandlungsdaten von *n* = 35.249 Fällen, die im Zeitraum 01.01.2016 bis 31.05.2018 im konservativen Bereich der ZNA der Universitätsmedizin Göttingen versorgt wurden. Hierbei wurden die aus dem Controlling zur Verfügung gestellten *Comma-separated-values*(csv)-Dateien von im Behandlungsverlauf resultierenden ICD-10-GM-Diagnosen mithilfe des Open-source-Statistikprogramms *RStudio* (Posit, Boston, USA) analysiert. Als Grundlage diente eine britische Erhebung bei > 10^6^ Patient*innen mit Definition des sog. *Hospital-Frailty-Risk-Scores* [4].

Zunächst wurden die häufigsten Diagnosen der stationären und der ambulanten Fälle separat ermittelt. Bei sehr vielen, differenzierten Subdiagnosen des ICD-10-GM-Systems wurden jeweils die 3‑stelligen ICD-Diagnosen verwendet.

Als Multimorbide wurden die Fälle definiert, bei denen ≥ 5 Diagnosen vorlagen. Alle weiteren wurden als Nichtmultimorbide weitergeführt. Um jeweils typische Diagnosen zu gewinnen, wurden alle Fälle in Multimorbide mit stationärer Behandlung (*n* = 15.652) und Multimorbide, die ambulant verblieben (*n* = 64), separiert. Restliche ambulante Multimorbide (*n* = 73) mit Ausschlussdiagnosen wurden nicht berücksichtigt.

Die über *RStudio* in 3‑stellige Subkategorien zusammengefassten Diagnosen, die am häufigsten vorkamen, wurden a priori in 112 *Subcluster *(Diagnosegruppen) zusammengefasst [4]. Risikofaktor*subcluster*, wie „Hypertonie“ (I10–I15) oder „Störung der Blutglukoseregulation“ (E10–E14, E16) wurden wegen allgemein bekannter Verknüpfung zwischen Risikoerkrankung und Folgeerkrankung ausgeschlossen. Um Kombinationen der vermehrt miteinander auftretenden *Subcluster* aufzuzeigen, wurden mithilfe des *RStudio*-Pakets *psych* und der Funktion *hclust* hierarchische Clusteranalysen durchgeführt. Dabei wurde für jeden Fall ein Vektor aus 112 *Subclustern* mit den binären Variablen „0“ (kein *Subcluster* zutreffend) bzw. „1“ (*Subcluster *zutreffend) erstellt. Anschließend wurden die binären Distanzen berechnet und nach der *Ward-D2*-Methode in Cluster fusioniert [4]. Hier stellt jeder Fall in sich anfangs ein eigenes Cluster dar und wird iterativ in weitere Cluster zusammengeführt. Mittels Dendrogramm, also einem Baumdiagramm, das die Ähnlichkeitsbeziehungen zwischen einer Gruppe von Entitäten darstellt, und nach abschließender Untersuchung der resultierenden Cluster auf Plausibilität wurde die Anzahl an Clustern ausgewählt, die die größte Steigerung der Heterogenität aufzeigte [4].

Über das Paket „*chron*“ erfolgte die Berechnung der Gesamt- bzw. ZNA-VWD stationärer MP nach Umwandlung der in den csv-Dateien hinterlegten Zeitangaben vom *Factor*- in das *Character*-Format. Die ZNA-VWD ambulanter MP wurde aus dem Informationssystem der ZNA ermittelt.

Um die statistische Signifikanz der Altersunterschiede zwischen den stationären und ambulanten MP – bei Normalverteilung der Daten im Q‑Q-Plot – zu ermitteln, wurde ein Welch-2-Stichproben-t-Test durchgeführt.

## Ergebnisse

### Charakteristika des Patientenkollektivs der hierarchischen Clusteranalyse

**Abb. 1:**
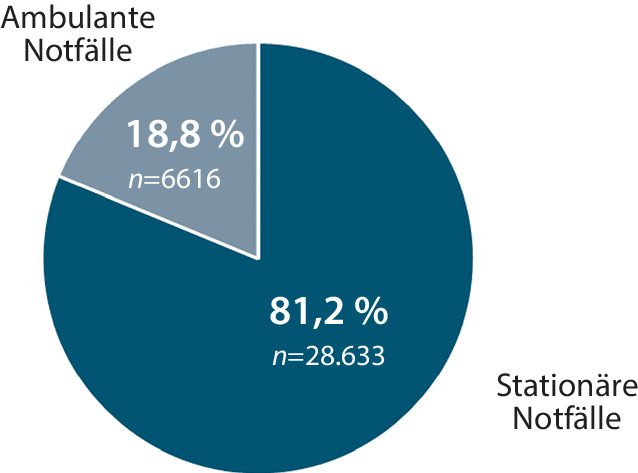
Anteil stationärer und ambulanter Notfälle im konservativen Bereich der zentralen Notaufnahme der Universitätsmedizin Göttingen im Zeitraum 01.01.2016 bis 31.05.2018

**Abb. 2:**
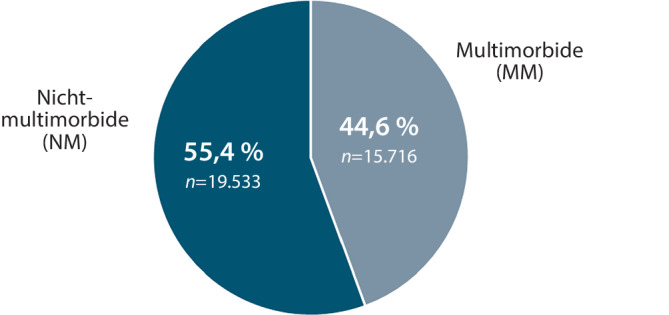
Analyse des Anteils multimorbider Notfallpatient*innen im Studienkollektiv

**Abb. 3:**
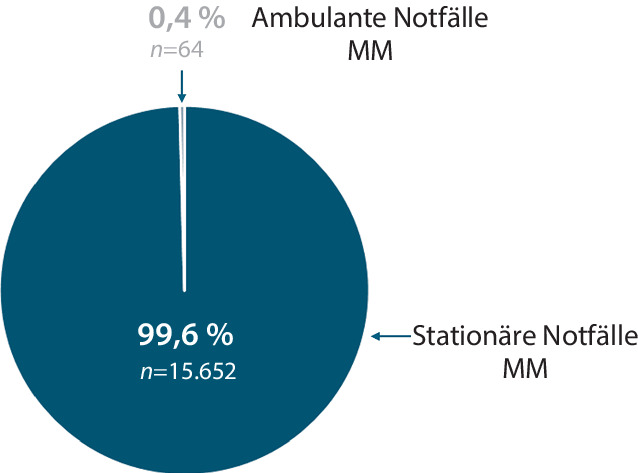
Disposition multimorbider Notfallpatient*innen im Studienzeitraum

In der monozentrischen, retrospektiven Clusteranalyse wurden in einem Zeitraum von 29 Monaten *n* = 35.249 Notfälle inkludiert; davon waren 53 % männlich und 47 % weiblich. Eine stationäre Aufnahme erfolgte in 81,2 % der Notfälle, 18,8 % verblieben ambulant (Abb. 1). Die Kriterien der MM waren in 44,6 % (*n* = 15.716) dieser Notfälle erfüllt (Abb. 2). Dabei wurden 99,6 % (*n* = 15.652) dieser multimorbiden Notfallpatient*innen stationär aufgenommen, nur 0,4 % (*n* = 64) konnten aus der Notaufnahme entlassen werden (Abb. 3).

Der Altersunterschied zwischen stationären und ambulant verbliebenen MP war hochsignifikant (stationäre MP 68,7/ambulante MP 60,8 Jahre; *p* < 0,001; 95 %-CI [3,73, 12,18]; t[63,5] = 3,76).

### Hierarchische Clusteranalyse der Diagnosekombinationen bei multimorbiden Patient*innen

**Abb. 4:**
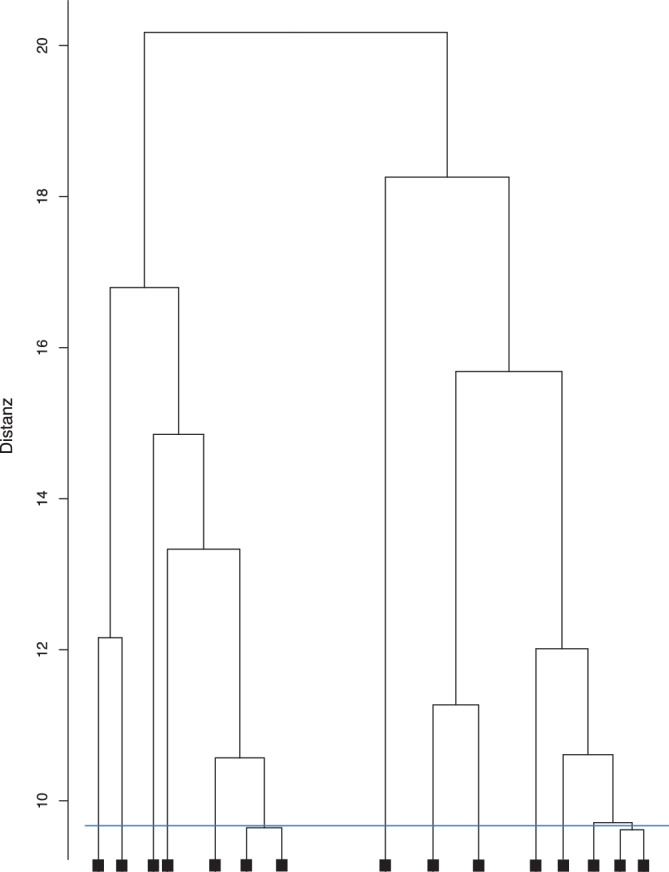
Ausschnitt aus dem Clusterdendrogramm stationärer, multimorbider Fälle. Die *blaue Linie* zeigt die Auswahl der 13 Cluster

**Abb. 5:**
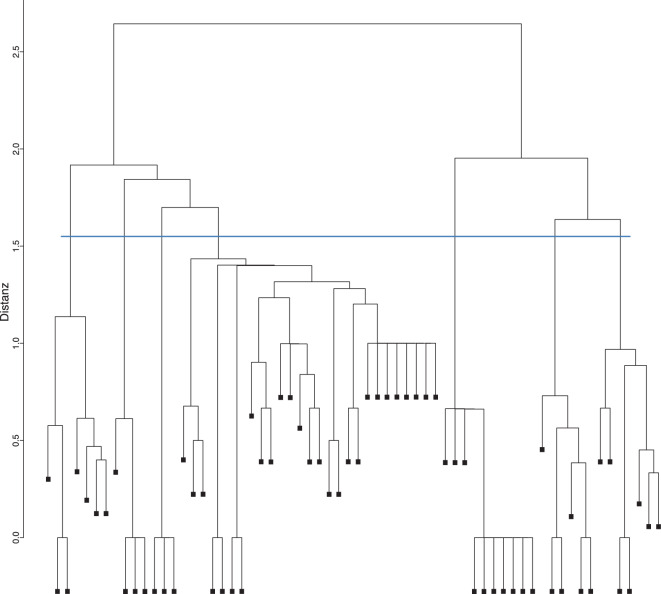
Clusterdendrogramm ambulanter, multimorbider Fälle. Die *blaue Linie* zeigt die Auswahl der 7 Cluster

Im Rahmen einer hierarchischen Clusteranalyse wurde ein Dendrogramm erstellt, das die hierarchische Anordnung der einzelnen Cluster verdeutlicht. Der Abstand einer horizontalen Linie, die 2 Cluster verbindet, zu der nächsten horizontalen Linie zeigt die Distanz jedes der Cluster zueinander. Je kleiner die Distanz eines Clusters zu einem anderen ist, desto ähnlicher sind sich diese. Visuell und nach inhaltlicher Überprüfung wurden für die stationären MP 13 Cluster (Abb. 4; Tab. 1) und für die ambulanten MP 7 Cluster (Abb. 5; Tab. 2) in den Diagnosekombinationen identifiziert.

**Tab. 1:** Tabellarische Übersicht der Diagnosencluster bei stationären multimorbiden Patient*innen

Cluster	Fallzahlen *n*	Clusterbezeichnung
1	428	Gravierende Zentralnervensystemsyndrome bei bekannten strukturellen zerebralen Erkrankungen
2	1265	Isolierte schwere Herzerkrankung
3	872	Schwere Pneumonien mit kardialer Erkrankung und akutem Nierenversagen
4	977	Deutlich symptomatische Infekte
5	629	Heterogene Diagnosekombinationen
6	1368	Schwere Pneumonien bei zerebralen Krankheiten
7	823	Zerebrale transitorische ischämische Attacke (TIA) bei Arrhythmie
8	922	Schwere Herzfunktionsstörung mit Begleiterkrankungen
9	3600	Bronchialerkrankung bei schwerer Herzerkrankung
10	843	Keine relevanten Diagnosekombinationen
11	1500	Systemische Infektionen mit diversen Diagnosekombinationen
12	1039	Zerebrale Erkrankungen und Arrhythmien
13	1388	Aspirationspneumonie und neurologische Erkrankungen

**Tab. 2:** Tabellarische Übersicht der Diagnosencluster bei ambulanten multimorbiden Patient*innen

Cluster	Fallzahlen *n*	Clusterbezeichnung
1	6	Hämatoonkologische Grunderkrankungen mit Begleiterkrankungen des Herzens und der Gefäße
2	7	Arrhythmien und Infekte der Atemwege
3	27	Pulmonale und hämatoonkologische Erkrankungen
4	4	Venen‑/Lymphsystemerkrankungen und endokrine Begleiterkrankungen
5	10	Hämatoonkologische Grunderkrankungen
6	7	Kardiovaskuläre und hämatoonkologische Erkrankungen
7	3	Solitäre Erkrankungen des ZNS

Die Analyse der Cluster mit den häufigsten Diagnosen ambulanter MP zeigte, dass darunter vorrangig das nichtfollikuläre Lymphom sowie die myeloische Leukämie aufgeführt waren. Einzelfallüberprüfungen ergaben, dass es sich bei den beiden größten ambulanten Clustern 3 (*n* = 27) und 5 (*n* = 10) um Patient*innen mit hämatoonkologischer Grunderkrankung handelte, bei denen es zu interkurrenten Erkrankungen, wie Pleuraerguss, COPD, Kandidose, akute Bronchitis oder endokrinologische Begleiterkrankungen, gekommen war. Somit wurden hämodynamisch stabile Patient*innen mit ohnehin zeitnah bestehendem ambulantem Termin nicht stationär aufgenommen. Auf Grundlage weiterer Überprüfungen ergaben sich Gründe für eine ambulante Behandlung anderer Patient*innen trotz ausgeprägter MM: So wurden z.B. bei Patient*innen aus dem Pflegeheim nach einer transitorischen ischämischen Attacke (TIA) mit Hinlauftendenzen im Rahmen der Demenzerkrankung vorrangig in der gewohnten Umgebung ambulant weiterbehandelt.

### Verweildauer in der ZNA und im Krankenhaus

Stationäre MP aus Cluster 3 und Cluster 11 verblieben am längsten im Krankenhaus (Gesamt-VWD 22,9 und 22,6 Tage). Hier standen vor allem Pneumonien, systemische Infektionen, kardiale Erkrankungen sowie das akute Nierenversagen im Vordergrund.

**Abb. 6:**
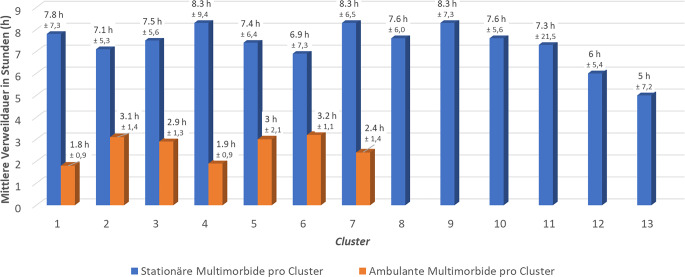
Verweildauer stationärer und ambulanter multimorbider Fälle in der ZNA

Bis zur weiteren Versorgung wiesen die stationären MP aus den Clustern 4, 7 und 9 die längste VWD in der ZNA auf (8,3 h; Abb. 6). Diese Cluster umfassten hauptsächlich bakterielle Infektionen, transitorische ischämische Attacken (TIA) oder andere ZNS-Syndrome (ohne Hirninfarkte) sowie chronische Atemwegserkrankungen. Bei den ambulanten MP betrug die maximale VWD in der ZNA höchstens 3,2 h (Cluster 6; Abb. 6). 100 % dieser Fälle wiesen Diagnosen auf, die den kardialen Erkrankungen zuzuordnen waren, 42,9 % davon den hämatologischen Malignomen.

## Diskussion

In der vorliegenden retrospektiven Studie wurde erstmalig eine strukturierte, hierarchische Clusteranalyse der Diagnosekombinationen von multimorbiden Notfallpatient*innen im konservativen Bereich einer ZNA der umfassenden Notfallversorgung in Deutschland durchgeführt. In der Analyse über einen 2‑jährigen Zeitraum ergab sich, dass 44,6 % der Notfälle die Kriterien der MM mit mehr als 5 Diagnosen erfüllten, hiervon mussten fast alle Fälle (99,6 %) stationär aufgenommen werden. Die stationären MP waren mit durchschnittlich 68,7 Jahren hochsignifikant älter als die ambulanten MP mit durchschnittlich 60,8 Jahren (*p* < 0,001). In der Literatur werden stationäre Aufnahmeraten von 20–60 % beschrieben. Allerdings wird auch die Abhängigkeit von Alter und Triagierung (> 60-Jährige mit dringlicher Behandlungsnotwendigkeit bei > 82 %) sowie Fachdisziplin (nichtchirurgische Notfälle mit höherer Rate als chirurgische) deutlich [7, 12]. Die hohe Konversionsrate in dem hier beschriebenen Patientenkollektiv ist einerseits bedingt durch das Altersspektrum und die Triagestufe der eingeschlossenen Patient*innen. Zudem ist die Universitätsmedizin Göttingen ein Krankenhaus der Maximalversorgung mit einem sehr großen Einzugsgebiet sowie eigener Klinik für Geriatrie und einem hohen Anteil von geriatrischen, schwerstkranken Notfallpatient*innen, d. h.: Insbesondere aus der Region werden MM mit hoher Triagestufe zugewiesen, die nachfolgend in den verschiedensten Fachdisziplinen der UMG versorgt werden.

Lai et al. [9] ermittelten demgegenüber im Jahr 2021, dass 95 % der stationären Patient*innen multimorbid waren, allerdings bei einer Definition von MM mit ≥ 2 chronischen Erkrankungen. Angesichts des medizinischen Fortschritts sind allerdings 2 aktive Diagnosen nicht selten.

Der „*claims-based frailty index*“ (CFI) u. a. über Mortalität, Beeinträchtigungen, Beweglichkeit und Sturzneigung bei Menschen ab 65 Jahren von Kim et al. (2018) stieg mit zunehmendem Alter [8]. Anders als bei diesem „*frailty index*“ zeigte sich in der vorliegenden Studie jedoch, dass MM auch bei Jüngeren relevant war.

Bei den von Marengoni et al. im Jahr 2009 [11] in Cluster zusammengefassten Erkrankungsgruppen (*n* = 1099; Einschlussalter ≥ 77 Jahre) erfolgte die Diagnoseerhebung über Fragebögen, klinische Untersuchungen und Laborergebnisse. Durch die Übermittlung der großen Zahl nur an Diagnosen als csv-Dateien konnte dies in der vorliegenden Studie jedoch objektiv und effizient erfolgen.

In der hierarchischen Clusteranalyse der vorliegenden Studie wurden für die *n* = 15.652 stationären MP 13 *Cluster* und für die *n* = 64 ambulanten MP 7 Cluster identifiziert.

Die Wahl einer aussagekräftigen Anzahl an Clustern im Rahmen einer Clusteranalyse gilt allgemein als problematisch, da statistische Methoden bei weit gestreuten Datensätzen nicht immer eindeutige Ergebnisse liefern [17]. In der vorliegenden Studie wurde die Clusteranzahl deshalb mittels Dendrogramm ausgewählt. Lai et al. (2021) identifizierten mit der sog. Elbow-Methode 5 Cluster bei MP > 45 Jahren [9]. Dabei resultiert nach iterativen *K‑Means*-Clusteranalysen verschiedener Clusteranzahlen eine Graphik, in der es zu einem Knick in Form eines Ellenbogens kommt, an dem die Mittelpunkte der Cluster sich annähern [10]. Auch Lai et al. führten am Ende eine Plausibilitätsprüfung durch, um eine klinisch sinnvolle Clusteranzahl zu erhalten, und definierten am Ende 9 Cluster [9].

Die Studie von Gilbert et al. [4] zeigt im Vergleich zur vorliegenden Studie Gemeinsamkeiten und Unterschiede. Die Gruppe wählte 109 für *Frailty* charakteristische Diagnosen aus und errechnete durch multiple Regressionsanalysen und c‑Statistik einen Hospital Frailty Risk Score (*HFRS*). Zusätzlich berücksichtigten sie Aufenthaltsdauer und -kosten. Es zeigten sich Cluster aus Kataraktoperationen, chronischen und akuten Herz- und Lungen- sowie malignen Erkrankungen mit jeweils erhöhtem *HFRS*. Ein Vergleich der *Frailty*-typischen Diagnosen mit der vorliegenden Studie zeigte ebenfalls u. a. Vorliegen von Demenz, Delir, „anderen zerebrovaskulären Erkrankungen“, Aspirationspneumonien und Sepsis.

In der vorliegenden Studie umfasst *Cluster 5* der stationären Fälle ebenfalls heterogene Krankheitsbilder: Einige dieser Erkrankungen zeigen offensichtlich eine stationäre Aufnahmeindikation an (gastrointestinale Blutung, akutes Nierenversagen), andere nicht (Ernährungsmangel, Adipositas, periphere arterielle Verschlusskrankheit). Im ambulanten Cluster (*n* = 64) der vorliegenden Studie fanden sich im Wesentlichen hämatoonkologische Erkrankungen.

1987 entwickelten Charlson et al. den *Charlson Comorbidity Index* mit Aussagen über die Ein-Jahres-Mortalität bei 17 vordefinierten Komorbiditäten. Dieser wurde jedoch nur monodimensional bei 559 Patient*innen entwickelt und bei 685 Patientinnen mit Mammakarzinom validiert [3].

Die Reduktion der VWD der Patient*innen stellt eine wichtige prozessuale Maßnahme für den Patientenflow in der ZNA dar. Ältere Patient*innen verbleiben jedoch in der Regel länger in der ZNA und werden häufiger aufgenommen [2, 13, 14]. Die vorliegende Arbeit zeigt, dass auch jüngere MP eine längere VWD in der ZNA aufweisen – vor allem bei besonderen Diagnosekombinationen, was offensichtlich eine längere Überwachungszeit und erhöhten diagnostischen und therapeutischen Aufwand bedingt. Auch sind organisatorische Faktoren wie Bettenkapazitäten zu berücksichtigen. Es bestätigt sich hinsichtlich der stationären VWD, dass sowohl Grunderkrankungen, wie Immunerkrankungen oder hämatologische Malignome, als auch schwerwiegende Komplikationen, wie Pleuraergüsse oder das akute Nierenversagen, ausschlaggebend für einen längeren Aufenthalt sind. Bei Clustern mit geringer Gesamt-VWD zeigten sich eher zeitnah zu therapierende Erkrankungen wie das Vorhofflimmern, die koronare Herzkrankheit oder der akute Myokardinfarkt [15]. Insgesamt zeigte sich ein Zusammenhang zwischen längerer ZNA- sowie Gesamt-Krankenhaus-VWD und den für MM typischen *Subclustern*.

### Limitationen

Das Design der vorliegenden Studie war lediglich retrospektiv und monozentrisch. Darüber hinaus wurden nach Durchführung der strukturierten Ersteinschätzung und Zuordnung in den konservativen Bereich der zentralen Notaufnahmen hauptsächlich internistische und neurologische Diagnosen berücksichtigt. Des Weiteren ist zu berücksichtigen, dass im Rahmen der Aufnahme von Notfällen nicht regelhaft alle Vorbefunde vorliegen, sodass sich hieraus Limitationen für die Identifikation von MM-Patient*innen ergeben können. Trotz der geringen Zahl der ambulanten Fälle wird im Sinne einer orientierenden Untersuchung ein Zusammenhang zwischen MM und Indikation einer stationären Behandlung deutlich.

## Ausblick

Mit weiteren, multizentrischen Studien zur hierarchischen Clusteranalyse von Diagnosekombinationen bei multimorbiden Notfallpatient*innen könnte schließlich die Entwicklung eines MM-Scores erfolgen. Damit wären MP mit erhöhtem Risiko für eine längere ZNA- oder Krankenhaus-VWD zeitnah in der ZNA zu identifizieren. Ergänzend zu aktuellen Kontextfaktoren wäre auch die Einbettung in den Katalog der ambulant durchführbaren Operationen (AOP) zur Begründung einer stationären Aufnahme möglich. Die Limitation der Identifikation von MM bereits in der Notaufnahme könnte durch die zukünftige Einführung der elektronischen Patientenakte behoben werden.

## Fazit für die Praxis

Spezifische, MM-typische und operationalisierbare Diagnosencluster sind geeignet, frühzeitig in einer ZNA eine stationäre Aufnahmenotwendigkeit anzuzeigen. Dadurch könnten Prozesse in der ZNA verkürzt, unnötige Aufnahmen reduziert und die Behandlungskosten verringert werden. Dies setzt eine Evaluation der Ergebnisse der hierarchischen Clusteranalyse dieser Pilotstudie in weiteren klinischen Studien voraus.

## Data Availability

Die erhobenen Datensätze können auf begründete Anfrage in anonymisierter Form beim korrespondierenden Autor angefordert werden. Die Daten befinden sich auf einem Datenspeicher im Studienzentrum der Zentralen Notaufnahme der Universitätsmedizin Göttingen
